# Wnt/β‐catenin signaling pathway inhibitors, glycyrrhizic acid, solanine, polyphyllin I, crocin, hypericin, tubeimoside‐1, diosmin, and rutin in medicinal plants have better binding affinities and anticancer properties: Molecular docking and ADMET study

**DOI:** 10.1002/fsn3.3405

**Published:** 2023-05-04

**Authors:** Chukwuebuka Egbuna, Kingsley C. Patrick‐Iwuanyanwu, Eugene N. Onyeike, Chukwuemelie Zedech Uche, Uchenna Petronilla Ogoke, Muhammad Riaz, Ebube Nnamdi Ibezim, Johra Khan, Kamoru A. Adedokun, Sikiru O. Imodoye, Ibrahim O. Bello, Chinaza Godswill Awuchi

**Affiliations:** ^1^ Africa Centre of Excellence in Public Health and Toxicological Research (ACE‐PUTOR) University of Port‐Harcourt Port Harcourt Nigeria; ^2^ Department of Biochemistry, Faculty of Science University of Port Harcourt Port Harcourt Nigeria; ^3^ Department of Biochemistry, Faculty of Natural Sciences Chukwuemeka Odumegwu Ojukwu University Uli Nigeria; ^4^ Department of Medical Biochemistry and Molecular Biology, Faculty of Basic Medical Sciences University of Nigeria Nsukka Nigeria; ^5^ Biostatistics and Computation Unit, Department of Mathematics and Statistics University of Port Harcourt Port Harcourt Nigeria; ^6^ Department of Allied Health Sciences University of Sargodha Sargodha Pakistan; ^7^ Department of Medical Laboratory Sciences, College of Applied Medical Sciences Majmaah University Al Majmaah Saudi Arabia; ^8^ Health and Basic Sciences Research Center Majmaah University Al Majmaah Saudi Arabia; ^9^ Department of Immunology Roswell Park Comprehensive Cancer Center Buffalo New York USA; ^10^ Department of Oncological Sciences, Huntsman Cancer Institute University of Utah Salt Lake City Utah USA; ^11^ Department of Biological Sciences Southern Illinois University Edwardsville Edwardsville Illinois USA; ^12^ School of Natural and Applied Sciences Kampala International University Kampala Uganda

**Keywords:** ADMET, anticancer drugs, β‐catenin, molecular docking, Wnt signaling pathways

## Abstract

Wnt/β‐catenin signaling pathway plays a role in cancer development, organogenesis, and embryogenesis. The abnormal activation promotes cancer stem cell renewal, proliferation, and differentiation. In the present study, molecular docking simulation and ADMET studies were carried out on selected bioactive compounds in search of β‐catenin protein inhibitors for drug discovery against cancer. Blind docking simulation was performed using PyRx software on Autodock Vina. β‐catenin protein (PDB ID: 1jdh) and 313 bioactive compounds (from PubChem database) with selected standard anticancer drugs were used for molecular docking. The ADMET properties of the best‐performing compounds were calculated using SwissADME and pkCMS web servers. The results obtained from the molecular docking study showed that glycyrrhizic acid, solanine, polyphyllin I, crocin, hypericin, tubeimoside‐1, diosmin, and rutin had the best binding interactions with β‐catenin protein based on their binding affinities. Glycyrrhizic acid and solanine had the same and lowest binding energy of −8.5 kcal/mol. This was followed by polyphyllin I with −8.4 kcal/mol, and crocin, hypericin, and tubeimoside‐1 which all had a binding energy of 8.1 kcal/mol. Other top‐performing compounds include diosmin and rutin with binding energy of −8.0 kcal/mol. The ADMET study revealed that the following compounds glycyrrhizic acid, solanine, polyphyllin I, crocin, hypericin, tubeimoside‐1, diosmin, rutin, and baicalin all violated Lipinski's rule of 5 which implies poor oral bioavailability. However, based on the binding energy score, it was suggested that these pharmacologically active compounds are potential molecules to be tested against cancer.

## Graphical abstract

Natural products have been known to have important therapeutic properties. Glycyrrhizic acid, solanine, polyphyllin I, crocin, hypericin, tubeimoside‐1, diosmin, and rutin could serve as scaffolds or leads for new anticancer therapy/drug.

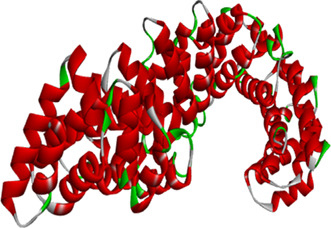

## 1 INTRODUCTION

The term Wnt is a portmanteau word formed from the terms Wingless and Int‐1. In animals, they are extremely evolutionarily conserved, meaning that they have similarities in all species of animals (Catalano et al., 2020; Söderholm & Cantù, 2020; Steinhart & Angers, 2018). The abnormality in Wnt/β‐catenin signaling pathway promotes cancer stem cell renewal, proliferation, and differentiation, playing important roles in carcinogenesis and therapeutic response (Zhang & Wang, 2020). Three Wnt signaling pathways are categorized, which include the “canonical Wnt signaling pathway,” the “noncanonical Wnt/calcium pathway,” and the “noncanonical planar cell polarity pathway.” All these pathways are usually activated by Wnt‐protein ligand binding to frizzled (Fz) family receptors, which transfer the biological signals to the disheveled proteins in the cells. The canonical Wnt pathway results in gene transcription regulation, and is believed to be partly regulated negatively by the SPATS1 gene (Steinhart & Angers, 2018). The pathway of noncanonical planar cell polarity is responsible for regulating the cytoskeleton that determines the cell shape. The pathway of noncanonical Wnt/calcium is responsible for regulating calcium in the cells. The canonical and noncanonical categories differ in that the noncanonical pathway operates independently of the protein β‐catenin while the canonical pathway involves β‐catenin (Chae & Bothwell, 2018; Söderholm & Cantù, 2020). The clinical significance of the pathway of noncanonical Wnt/calcium was shown by the mutations that resulted in several diseases, such as type II diabetes, glioblastoma, and breast and prostate cancer (Fatima et al., 2021). Recently, there is a report of the first successful use of the inhibitors of the Wnt pathway of disease in mouse models (Ng et al., 2019).

Wnt is made up of several secreted lipid‐modified signaling glycoproteins family which are 350‐ to 400‐amino‐acid units in length. Palmitoleoylation of a single completely conserved residue of serine is all Wnts' lipid modification (Hannoush, 2015). Palmitoleoylation is essential as it is a requirement for the binding of Wnt to its carrier protein WLS (Wntless) to facilitate its transportation to plasma membranes for secreting purposes; it makes protein of Wnt to bind its Fz Wnt protein receptors and undertake glycosylation, attaching a carbohydrate so as to ensure appropriate secretion (Hosseini et al., 2019; Janda et al., 2012; Nygaard et al., 2021; Yu et al., 2014). “In the Wnt signaling, these proteins function as ligands that activate the various Wnt signaling pathways through autocrine and paracrine routes” (Fatima et al., 2021). The proteins are extremely conserved in all species. They are found in *Drosophila*, zebrafish, *Xenopus*, humans, mice, etc. (Hosseini et al., 2019; Janda et al., 2012; Yu et al., 2014).

Wnt signaling starts when the “N‐terminal cysteine‐rich extracellular domain of a Fz family receptor” is bound by a Wnt protein (Chae & Bothwell, 2018). These Fz family receptors span the plasma membranes by sevenfold and constitute a different “G‐protein coupled receptors” (GPCRs) family (Awuchi, 2023; Kramer et al., 2017; Li et al., 2021; Takahashi et al., 2017). Nevertheless, co‐receptors may be required to encourage Wnt signaling along with the interactions between the Fz receptor and the Wnt proteins. Examples include ROR2, receptor tyrosine kinase (RTK), and “lipoprotein receptor‐related protein (LRP)‐5/6.” Upon the receptor activation, disheveled (Dsh), a cytoplasmic phosphoprotein, is sent a signal (Takahashi et al., 2017; Kramer et al., 2017). This signal sent to Dsh is transmitted through direct interaction between disheveled and Fz. Disheveled proteins occur in all organisms, which all have a share of highly conserved domains of protein; including a carboxy‐terminal DEP, a central PDZ, and amino‐terminal DIX domains (Patel et al., 2019). “All the various domains are significant because after dishevelled, the Wnt signal may branch off into several pathways, with each pathway interacting with a separate combination of all domains” (Takahashi et al., 2017; Kramer et al., 2017).

In addition, Wnt signaling has been known to regulate several other signaling pathways which have no extensive elucidation yet. One of the pathways includes the biological interactions between GSK3 and Wnt. Wnt could inhibit GSK3 during cell growth so as to activate mTOR without the presence of β‐catenin. Also, Wnt can act as an mTOR‐negative regulator through tumor suppressor TSC2 activation, which is upregulated through the interaction of GSK3 and disheveled (Kuroda et al., 2013). Wnt makes use of CREB and PA to activate Myf5 and MyoD genes during myogenesis (Kuroda et al., 2013). It functions together with Src and Ryk in order to allow neuron repulsion regulation in axonal guidance. Several other signaling pathways are regulated by the Wnt (Kuroda et al., 2013; Malinauskas & Jones, 2014).

For ensuring appropriate functioning, there is continuous regulation of Wnt signaling at multiple points in the Wnt signaling pathways (Kramer et al., 2017; Malinauskas & Jones, 2014; Takahashi et al., 2017; Zhan et al., 2017). For example, Wnt proteins are palmitoylated via a process mediated by *porcupine* (protein), meaning that it aids the regulation once there is secretion of Wnt ligand by determining its full formation (Klemm & Joyce, 2015; Kramer et al., 2017; Sales et al., 2016; Takahashi et al., 2017). There is further control of the secretion with proteins (e.g., GPR177) and evenness disrupted and complexes including retromer complex. Once secreted, there can be prevention of the ligand from being in contact with its receptor via the binding of proteins including the stabilizers glypican 3 (GPC3) and Dally that inhibit diffusion. Both the GPC3 core protein and the heparan sulfate chains play role in the regulation of Wnt activation and binding for proliferation of cells in cancer cells (Gao et al., 2014, 2015, 2016; Ho & Kim, 2011; Li et al., 2018, 2019). Wnt recognizes a structure of heparan sulfate on GPC3 that has GlcNS6S and IdoA2S, and the Wnt binding to the glypican of heparan sulfate is enhanced by the “3‐O‐sulfation in GlcNS6S3S” (Gao et al., 2016; Gordon & Gordon, 2016; Minde et al., 2013). A domain rich in cysteine at the GPC3 N‐lobe has been acknowledged for forming a “hydrophobic groove” that binds to Wnt, such as phenylalanine‐41, which has interaction with Wnt (Kolluri & Ho, 2019; Li et al., 2019). Blocking the binding domain of Wnt with a nanobody known as HN3 could inhibit the activation of Wnt (Li et al., 2019). In this study, molecular docking simulation was carried out to determine β‐catenin inhibitors for drug discovery against cancer.

## 2 MATERIALS AND METHODS

### 2.1 Ligand curation and preparation

To curate phytochemicals that have been previously asserted to have anticancer effects, a literature search was done. Phytochemistry: An in silico and in vitro Update, edited by Kumar and Egbuna, and Phytochemistry, Volume 1: Fundamentals, Methods, and Applications by Egbuna et al. (2018) are a few of the materials consulted. Also, Drug Development for Cancer and Diabetes: A Path to 2030, edited by Saravanan et al. (2020), and Phytochemicals as Lead Compounds for New Drug Discovery, edited by Kumar and Egbuna (2019) were consulted. The NCBI PubChem database was used to retrieve the chemical structures (in 3D SDF) of 313 chemical compounds as well as traditional anticancer medications and their related CIDs. To convert 2D SDF files to 3D SDF formats, VeraChem LLC's VConf software was utilized, and ChemDraw Ultra 12.0 was used to depict substances that were not identified in the PubChem and ChemSpider databases (CambridgeSoft). Using the Open Babel software, all of the ligands were compressed into a single SDF file to make it simpler to integrate into the PyRx program (openbabel.org).

### 2.2 Protein preparation

**FIGURE 1:**
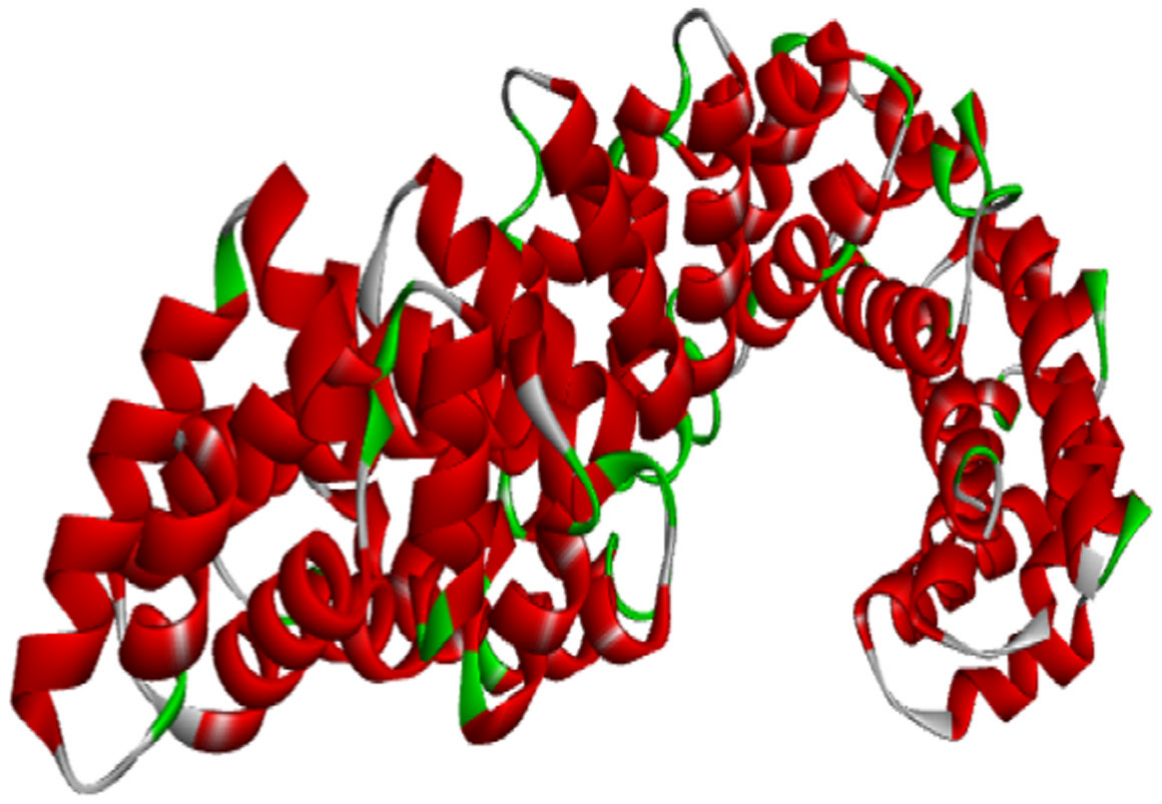
The 3D structure of β‐catenin protein (PDB ID: 1jdh) before docking.

The β‐catenin protein with PDB ID: 1jdh (Resolution: 1.90 Å) was obtained in .pdb format from the Protein Data Bank (https://www.rcsb.org/) and processed using BIOVIA Discovery Studio Visualizer 2021 v21.1.0.20298 (Dassault Systèmes: https://www.3ds.com) according to Qasaymeh et al. (2019). Water molecules and hetero atoms were removed during processing, whereas polar hydrogens were added. Only chain A was retained for blind docking. The 3D structure of β‐catenin protein prior to docking is shown in Figure 1.

### 2.3 Active site prediction

**FIGURE 2:**
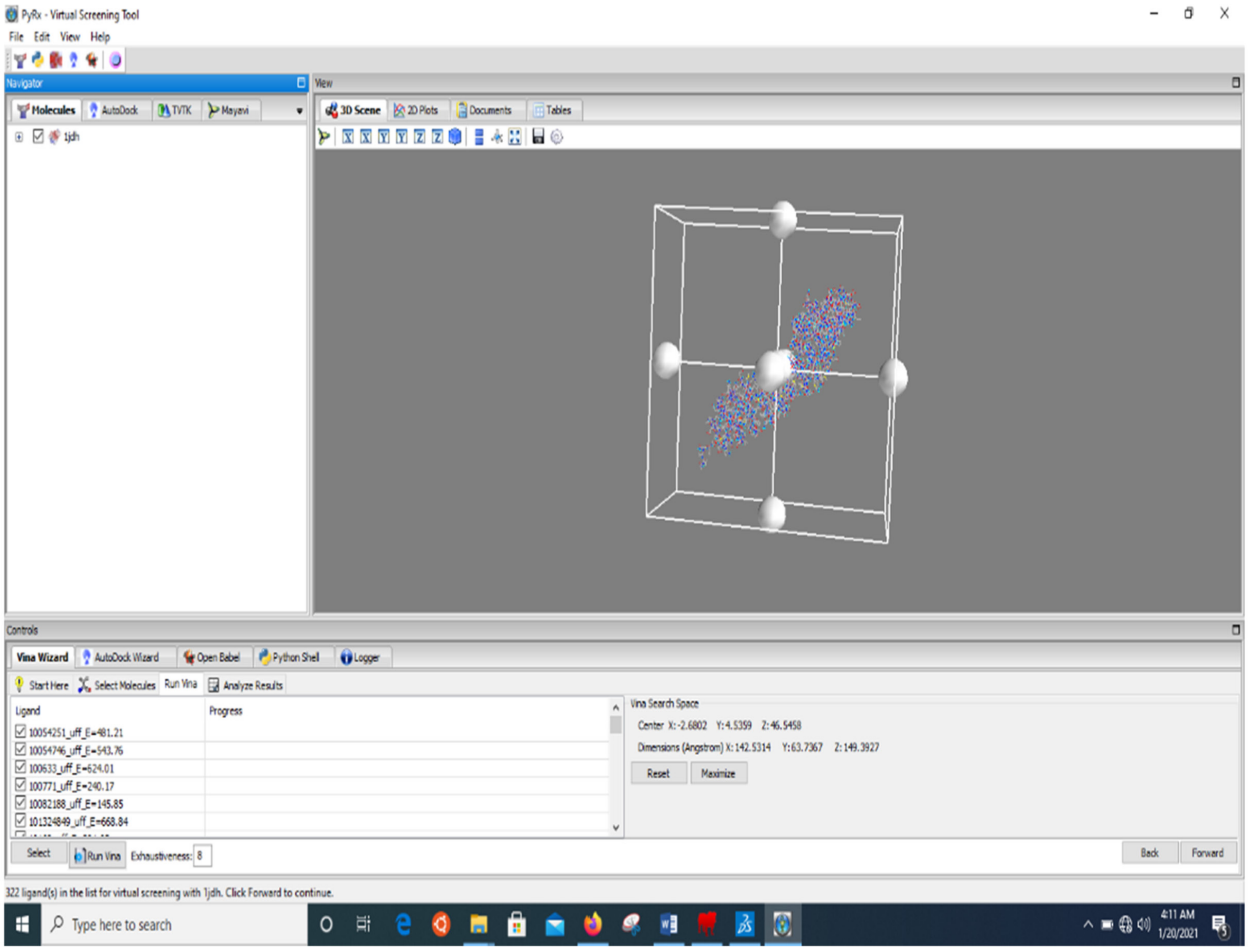
Positioning of grid box for blind docking.

Due to the scarcity of information about the active site of β‐catenin, blind docking was done on the protein surface in chain A using PyRx software by positioning the grid box as shown in Figure 2. The resolved center coordinate is as follows:

**Table:** 

Centre	*X*: −2.6802	*Y*: 4.5359	*Z*: 46.5458
Dimensions (Angstrom)	*X*: 142.5314	*Y*: 63.7367	*Z*: 149.3927

### 2.4 Molecular docking studies

According to Dallakyan and Olson (2015), molecular docking simulations were carried out using PyRx software version 0.8 (https://pyrx.sourceforge.io). PyRx is a program for high‐throughput virtual screening of compounds against protein targets using molecular docking simulations. By analyzing the binding energy of compounds in kcal/mol, it is possible to determine which substances have the best chances of forming a strong bond with a protein. In this work, the 3D SDF‐formatted ligands that had been generated and compressed were loaded into PyRx using the built‐in OpenBabel graphical user interface. The conjugate gradient approach was used to minimize energy using the universal force field (UFF), with a total number of steps set at 200. One update step was specified, followed by a stop step if the energy difference was less than 0.1 kcal/mol. Afterward, all ligands were changed into AutoDock ligands to reduce energy use (pdbqt). In order to prepare the protein for docking, it was loaded into PyRx and then changed to .pdbqt. Docking simulation was run with an exhaustiveness level of 8. The ligand with the highest binding affinity was identified as having the highest binding energy (which is most negative) (Prasanth et al., 2020). Using BIOVIA Discovery Studio, specific interactions of the optimum docking poses were shown. The docking protocol was validated.

### 2.5 Theoretical prediction of ADMET parameters

Following the completion of the docking simulation, the top‐ranked compounds (first 20 compounds plus common inhibitors) were transferred to SwissADME and pkCMS online servers in canonical SMILES format for toxicity and bioavailability prediction techniques such as Lipinski's rule of 5. SwissADME (http://www.swissadme.ch/) and pkCMS (http://biosig.unimelb.edu.au/pkcsm/prediction) are free online resources for predicting the pharmacokinetics, drug likeness, and medicinal chemistry friendliness of small compounds (Daina et al., 2017; Pires et al., 2015). Table 1 lists the significance and normal distribution of a few ADMET parameters chosen for this investigation (Egbuna et al., 2021).

**TABLE 1:** Acceptable range of selected ADMET properties.

Parameters	Significance	Acceptable range	References
SwissADME			
Physicochemical properties			
MW	Molecular weight (MW). MW is the mass of a given molecule. It is expressed in Daltons (Da or u), synonymous with g/mol.	Between 150 and 500 g/mol	Daina et al. (2017)
nHA	Number of heavy atoms (nHA)	20–70	Ghose et al. (1999)
HBA	HBA: No. of hydrogen bond acceptors. Represents total nitrogen and oxygen atoms (NorO)	<10	Lipinski et al. (2001) and Daina et al. (2017)
HBD	HBD: No. of hydrogen bond donors. Represents total N–H and O–H bonds (NH or OH). High HBD can lead to low‐fat solubility.	<5	Lipinski et al. (2001) and Daina et al. (2017)
MR	MR (molecular refractivity) is a measure of the total polarizability of a mole of a substance.	40–130	Ghose et al. (1999)
TPSA	TPSA (topological polar surface area). TPSA denotes a compound's capacity to penetrate cell membranes. A TPSA greater than 140 Å^2^ is deemed unsatisfactory.	Between 20 and 130 Å^2^	Daina et al. (2017)
ROTBs	ROTBs: Number of rotatable bonds. Denotes flexibility	<9	Daina et al. (2017)
Lipophilicity			
Log *P*_O/W_	Log *P*_O/W_: is the consensus arithmetic mean of five proposed methods.	−0.7 and +5.0 for XLOGP3	Daina et al. (2017)
Water solubility			
Log *S*	Log *S* (ESOL): Decimal logarithm of the molar solubility in water.	<6	Daina et al. (2017)
Drug‐likeness			
Lipinski's rule of 5	Lipinski et al. (2001) state that an oral medication candidate must not breach more than one of the following requirements (as stated above): 1. There should not be more than five hydrogen bond donors (the total number of nitrogen–hydrogen and oxygen–hydrogen bonds). 2. There should not be more than 10 hydrogen bond acceptors (all nitrogen or oxygen atoms). 3. The molecular weight should be <500 Daltons. 4. The octanol–water partition coefficient (log *P*) must not be more than 5. 5. TSPA should be no more than 40 Å^2^	YES, if there is any violation and NO if there is none.	Daina et al. (2017)
pkCMS			
Absorption			
Caco‐2	This foretells the absorption rate of oral drugs in the intestinal mucosa	High if >0.90	Pires et al. (2015)
Intestinal absorbance	This predicts the % to be absorbed	Poor if <30%	Pires et al. (2015)
P‐gp I and II inhibitors	Xenobiotics are eliminated from cells through the ATP‐binding cassette transporter P‐glycoprotein (P‐gp). This model foretells whether or not a substance will likely act as an inhibitor of it.	Inhibits P‐gp I or II, both or none	Pires et al. (2015)
Distribution			
BBB	BBB (Blood–brain barrier). Necessary to know the behavior of exogenous compounds as regards BBB.	Compounds with LogBB >0.3 can cross the BBB while those with logBB < −1 would be poorly distributed	Pires et al. (2015)
CNS	The CNS permeability model evaluates blood–brain permeability of surface area product (logPS)	Compounds with logPS > −2 can penetrate the CNS while logPS < −3 cannot	Pires et al. (2015)
Metabolism			
CYP2D6	Cytochrome P450 detoxifies drugs and xenobiotics. CYP2D6 metabolizes ≈25% of drugs (Wang et al., 2009)	YES or NO if metabolized by CYP2D6	Pires et al. (2015)
CYP3A4	CYP3A4 metabolizes ≈ half of 60% of drugs credited to cytochrome P450 (Zanger and Schwab, 2013)	YES or NO if metabolized by CYP3A4	
Excretion			
TC	TC (total clearance) considers both hepatic and renal clearance.	Measured in log (ml/min/kg)	Pires et al. (2015)
Toxicity			
Ames	Ames predicts mutagenicity.	YES or NO	Pires et al. (2015)
MRTD	MRTD (maximum recommended tolerated dose) provides an estimate of the toxic threshold of chemicals.	MRTD ≤0.477 is low while ≥0.477 is high	Pires et al. (2015)
LD_50_	LD_50_: Dose capable of causing death in 50% of test animals.	Predicts LD_50_ in mol/kg	Pires et al. (2015)
Hepatotoxicity	This predicts liver injury.	YES or NO	Pires et al. (2015)

## 3 RESULTS AND DISCUSSION

### 3.1 Molecular docking

Wnt signaling pathway is a collection of signal transduction pathways that play a role in development, organogenesis, and embryogenesis as it concerns morphogenesis and cellular movements (Gruszka et al., 2019). Body axis patterning, cell fate determination, cell proliferation, and cell migration are among the embryonic activities it regulates. Wnt is implicated in carcinogenesis. It is found to be upregulated in acute myeloid leukemia and significant in the maintenance of leukemic stem cells (Gruszka et al., 2019). In solid and hematological tumors, Wnt signaling targeting is a strategy that is being investigated (Gruszka et al., 2019). The clinical significance of this pathway has also been shown by mutations that result in a number of disorders, including type II diabetes, glioma, breast and prostate cancer, and others (Logan & Nusse, 2004). In AML, there are a number of Wnt molecules with prognostic values. They include: β‐catenin, LEF‐1, phosphorylated‐GSK3, AXIN2, PPARD, CXXC5, PCDH17, PSMD2, PTK7, XPNPEP, RUNX1, LLGL1, and sFRP2 (Gruszka et al., 2019). In AML, it was established that Wnt pathway activation leads to a high concentration of β‐catenin, which causes significant effect on disease's prognosis. Poor event‐free survival (EFS), shorter overall survival (OS), and increased clonogenic activity were shown to be predicted by high levels of β‐catenin expression in AML revealed in Western blot analysis (Ysebaert et al., 2006). In other words, inhibiting β‐catenin could be a life‐saving measure in AML.

**FIGURE 3:**
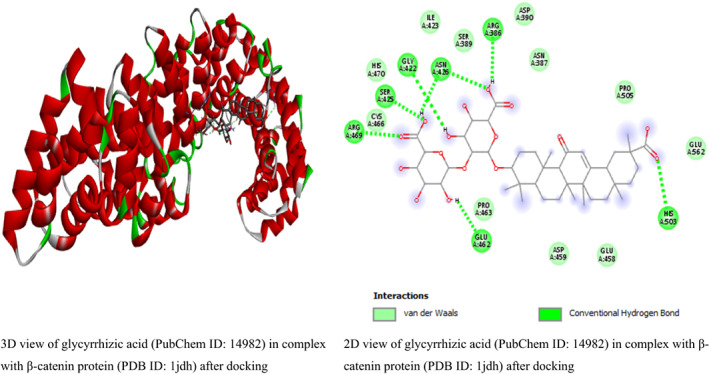
The 3D and 2D views of β‐catenin protein interactions with glycyrrhizic acid after docking.

**FIGURE 4:**
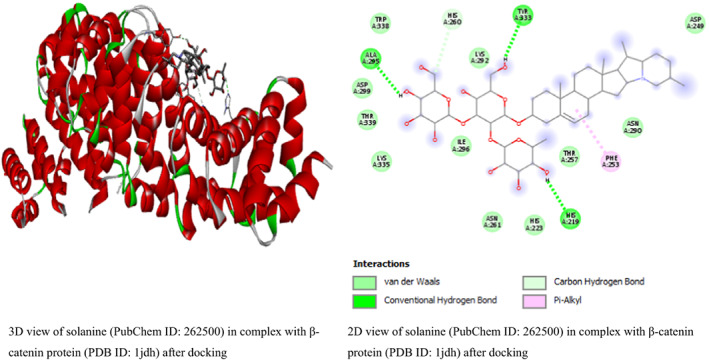
The 2D view of β‐catenin protein interactions with solanine after docking.

**FIGURE 5:**
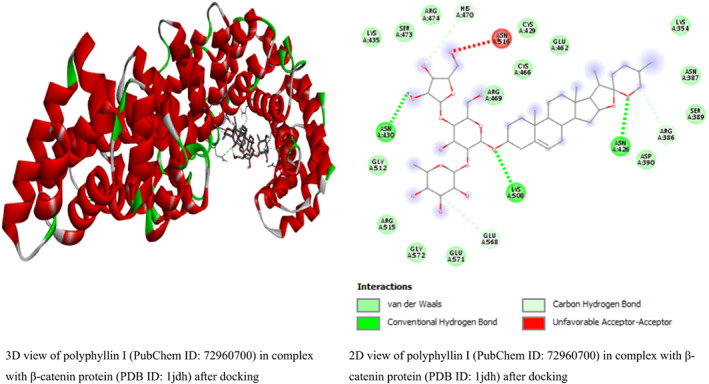
The 2D view of β‐catenin protein interactions with polyphyllin I after docking.

**FIGURE 6:**
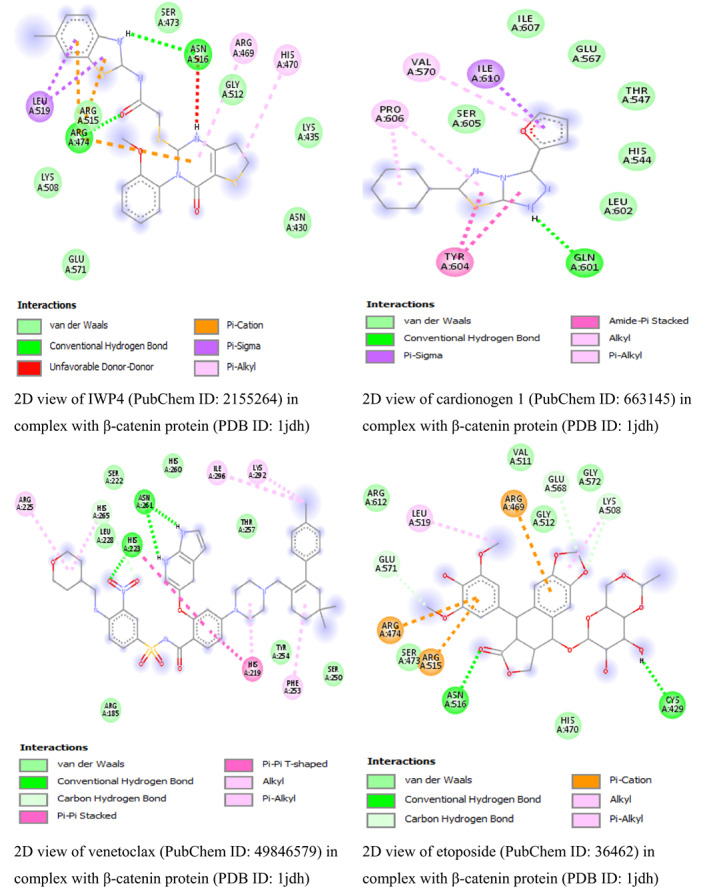
The 2D view of interactions of β‐catenin with top runners after docking.

In this study, the result from β‐catenin molecular docking study (Table 2) revealed that glycyrrhizic acid and solanine came top with the same binding energy of −8.5 kcal/mol as possible inhibitors of β‐catenin. To the best of our knowledge, this is the first study linking β‐catenin to glycyrrhizic acid and solanine which may open a new door for drug discovery or offer explanatory mechanism behind the anticancer activity of glycyrrhizic acid and solanine. This result is in line with the study by Zuo et al. (2022) who found that glycyrrhizic acid has strong activity against colorectal cancer cells via SIRT3 inhibition. However, one drawback of glycyrrhizic acid is that it is not readily absorbed in the intestine (Roohbakhsh et al., 2016). For solanine, many studies have linked it to having anticancer activity. A study by Lin et al. (2020) found that solanine inhibited cell proliferation and migration by epithelial–mesenchymal transition and matrix metalloproteinases inhibition in acetylcholine‐treated Hep G2 cells. This is a positive attribute of solanine. However, solanine as a single agent, although poorly absorbed in the intestine, has been reported to be very toxic (Ordóñez‐ Vásquez et al., 2019), especially to the liver. The result for glycyrrhizic acid and solanine is followed by the binding energies (in kcal/mol) of polyphyllin I (−8.4), crocin (−8.1), hypericin (−8.1), tubeimoside‐1 (−8.1), diosmin (−8), rutin (−8), baicalin (−7.9), and tomatidine (−7.9). The binding energies are better compared to the binding energies of β‐catenin standard inhibitors IWP4 (−7.1 kcal/mol) and cardionogen 1 (−6.3 kcal/mol). However, venetoclax with binding energy of −8.5 kcal/mol, and some standard anticancer drugs such as etoposide (−8 kcal/mol), teniposide (−8 kcal/mol), and (−)‐rapamycin (−7.7 kcal/mol) performed better than the standard Wnt/β‐catenin pathway inhibitors and some natural compounds (Table 2). The 3D and 2D structures of the first top runners were presented in Figure 3. The result indicates that glycyrrhizic acid in complex with β‐catenin formed eight conventional hydrogen bonds at GLY422, SER425, ASN426, CYS466, ARG469, GLU462, HIS503, and ARG386 amino acid residues. The rest amino acid residues were found to only form van der Waals bonds. For solanine (Figure 6), three conventional hydrogen bonds were formed at ALA295, TYR333, and HIS219 amino acid residues while carbon hydrogen bond and pi‐alkyl bonds were formed at HIS260 and PHE253 amino acid residues, respectively. For polyphyllin I, three conventional hydrogen bonds were formed with amino acid residues ASN430, LYS508, and ASN426. Also, three carbon–hydrogen bonds were formed at HIS470, GLU568, and ARG386. The aceptor‐aceptor interaction formed with ASN516 was unfavorable. The result shows that β‐catenin–IWP4 complex formed two conventional hydrogen bonds at amino acid residue ASN516 and ARG474. Pi‐alkyl (at ARG469 and HIS470 amino acid residues) and pi‐sigma bonds (at LEU519) were formed. For cardionogen I, the following bonds were found: conventional hydrogen bonds (GLN601), alkyl or pi‐alkyl bonds (PRO606 and VAL570), amide‐pi‐stacked (TYR604), and pi‐sigma (ILE610) bonds. In Figure 4, the 2D view of β‐catenin protein interactions with solanine after docking can be seen. The 2D view of β‐catenin protein interactions with polyphyllin I after docking is shown in Figure 5. In Figure 6, the 2D view of the molecular interactions of β‐catenin and standard inhibitors was presented.

**TABLE 2:** Molecular docking scores of β‐catenin protein inhibitors.

S/No	Compound name	PubChem ID	Binding energy (kcal/mol)	Sources
1.	Glycyrrhizic acid	14982	−8.5	Licorice (root extract) and *Glycyrrhiza glabra* (Fabaceae)
2.	Solanine	262500	−8.5	Nightshade family, e.g., genus Solanum, e.g., potato, tomato, and eggplants
3.	Polyphyllin I	72960700	−8.4	*Paris polyphylla*
4.	Crocin	5281233	−8.1	Flowers of crocus and gardenia, saffron
5.	Hypericin	3663	−8.1	Genera *Hypericum* (Saint John's wort)
6.	Tubeimoside‐1	51346132	−8.1	*Bolbostemma paniculatum*
7.	Diosmin	5281613	−8.0	Citrus fruits (oranges and lemons) and peel extracts, hyssop, and figwort
8.	Rutin	5280805	−8.0	Citrus leaves (orange and lime), tomato, green tea, fenugreek, and olive
9.	Baicalin	64982	−7.9	Plants in genus *Scutellaria* and in *Oroxylum indicum*
10.	Tomatidine	65576	−7.9	Stems and leaves of tomato plants, and in the fruits at low concentrations
11.	Ginkgolide‐C	9867869	−7.8	*Ginkgo biloba* L.
12.	Myricetin	5281672	−7.8	Fruits (e.g., oranges), tomatoes, nuts, berries, and tea
13.	β‐Sitosterol	222284	−7.7	Vegetable oil, nuts, avocados, and salad
14.	Glabridin	124052	−7.7	Licorice (root extract) and *Glycyrrhiza glabra*
15.	Ursolic acid	64945	−7.7	Apples, berries, elder flowers, peppermint, lavender, and oregano
16.	Withanolide	53477765	−7.7	Nightshade plant family, e.g., *Datura*, *Solanum*, *Withania*, and *Jaborosa*
17.	Quercetin	5280343	−7.6	Apples, honey, berries, onions, red grapes, citrus fruits, and green vegetables
18.	Tetrandine	73078	−7.6	*Stephania tetrandra* and species of Menispermaceae
19.	Vicenin‐2	442664	−7.5	Sweet oranges, *Ocimum sanctum*, buckwheats, and fenugreeks
20.	Vitexin	5280441	−7.5	Leaves of *Phyllostachys nigra* (Bamboo), passion flower, and pearl millet
**Standard β‐Catenin Drugs**				**Target**
1.	IWP4	2155264	−7.1	β‐Catenin inhibitor
2.	Cardionogen 1	663145	−6.3	β‐Catenin inhibitor
**Other top‐performing anticancer drugs**				
3.	Venetoclax	49846579	−8.5	Bcl‐2 inhibitor
4.	Etoposide	36462	−8.0	Anticancer drug (semisynthetic derivative of podophyllotoxin)
5.	Teniposide	452548	−8.0	Anticancer drug (podophyllotoxin derivative)
6.	(−)‐Rapamycin	5,284,616	−7.7	mTOR inhibitor

### 3.2 Drug‐likeness and ADMET prediction

Absorption, distribution, metabolism, excretion, and toxicity collectively go by the abbreviation ADMET. To foretell a molecule's potential behavior both inside and outside the body, theoretical prediction of ADMET was performed. This test is helpful in providing medicinal chemists or drug developers with an insight into the drug‐likeness of molecules in order to avoid end‐stage test failures. The drug‐likeness and ADMET features of the front‐runners derived from the molecular docking result were determined using SwissADME and pkCMS web servers. The drug‐likeness result (Table 3) shows that the first nine performing compounds (glycyrrhizic acid, solanine, polyphyllin I, crocin, hypericin, tubeimoside‐1, diosmin, rutin, and baicalin) all violated the Lipinski's rule of 5. However, the following compounds, tomatidine, ginkgolide‐C, myricetin, β‐sitosterol, glabridin, ursolic acid, withanolide, and quercetin did not violate Lipinski's rule of 5. According to the rule, a potential compound will be orally bioavailable if it did not violate more than one rule (Kumar & Egbuna, 2019; Lipinski et al., 2001; Rudrapal & Egbuna, 2022).

**TABLE 3:** Physicochemical and drug‐likeness properties of front‐runners and standard drugs by SwissADME.

S/N	Selected front‐runners	Physicochemical properties							Lipophilicity	Water solubility	Drug‐likeness
		MW	nHA	HBA	HBD	MR	TPSA	RB	Log *P*_O/W_	Log *S*	Lipinski's rule of 5
1.	Glycyrrhizic acid	822.93	58	16	8	202.84	267.04	7	2.25	−6.24	No; 3 violations: MW > 500, NorO > 10, NHorOH > 5
2.	Solanine	868.06	61	16	9	222.19	240.69	8	−0.23	−5.83	No; 3 violations: MW > 500, NorO>10, NHorOH>5
3.	Polyphyllin I	855.02	60	16	8	211.61	235.68	8	0.85	−5.53	No; 3 violations: MW > 500, NorO > 10, NHorOH > 5
4.	Crocin	976.96	68	24	14	227.19	391.20	20	−5.23	−3.01	No; 3 violations: MW > 500, NorO > 10, NHorOH > 5
5.	Hypericin	504.44	38	8	6	144.52	155.52	0	5.79	−6.99	No; 2 violations: MW > 500, NHorOH > 5
6.	Tubeimoside‐1	1347.49	94	29	15	318.76	456.19	4	−2.51	−6.93	No; 3 violations: MW > 500, NorO > 10, NHorOH > 5
7.	Diosmin	608.54	43	15	8	143.82	238.20	7	−1.09	−3.51	No; 3 violations: MW > 500, NorO > 10, NHorOH > 5
8.	Rutin	610.52	43	16	10	141.38	269.43	6	−1.69	−3.30	No; 3 violations: MW > 500, NorO > 10, NHorOH > 5
9.	Baicalin	446.36	16	11	6	106.72	187.12	4	0.14	−3.41	No; 2 violations: NorO > 10, NHorOH > 5
10.	Tomatidine	415.65	30	3	2	127.70	41.49	0	4.99	−6.33	Yes; 1 violation: MLOGP > 4.15
11.	Ginkgolide‐C	440.40	31	11	4	94.45	169.05	1	−2.40	−1.65	Yes; 1 violation: NorO > 10
12.	Myricetin	318.24	23	8	6	80.06	151.59	1	1.69	−3.01	Yes; 1 violation: NHorOH > 5
13.	β‐Sitosterol	576.85	41	6	4	165.61	99.38	9	5.85	−7.70	Yes; 1 violation: MW > 500
14.	Glabridin	324.37	24	4	1	93.25	58.92	1	3.89	−4.61	Yes; 0 violation
15.	Ursolic acid	456.70	33	3	2	136.91	57.53	1	7.09	−7.23	Yes; 1 viol.: MLOGP > 4.15
16.	Withanolide	470.60	34	6	2	127.53	96.36	2	3.50	−4.59	Yes; 0 violation
17.	Quercetin	302.24	22	7	5	78.03	131.36	1	1.99	−3.16	Yes; 0 violation
Standard drugs in clinical use/trial											
IWP‐4		496.62	33	5	1	134.60	164.95	7	4.35	−5.92	Yes; 0 violation
Cardionogen 1		274.34	19	4	0	73.25	84.46	2	3.49	−3.94	Yes; 0 violation
Other anticancer drugs											
Venetoclax (ABT‐199)		868.44	61	9	3	246.70	183.09	14	8.79	−9.78	No; 2 viols: MW > 500, NorO > 10
Etoposide		588.56	42	13	3	139.11	160.83	5	1.01	−3.75	No; 2 violations: MW > 500, NorO > 10
Teniposide		656.65	46	13	3	156.66	189.07	6	2.10	−4.57	No; 2 violations: MW > 500, NorO > 10
Rapamycin		914.17	65	13	3	253.68	195.43	6	5.80	−8.90	No; 2 violations: MW > 500, NorO > 10

*Note*: Log *P*_O/W_ (WLOGP).

Abbreviations: HBA, number of hydrogen bond acceptors; HBD, number of hydrogen bond donors; MR, Molecular refractivity; MW, molecular weight (Dalton); nHA, number of heavy atoms; RB, number of rotatable bonds; TPSA, topological polar surface area (Å^2^).

For the ADMET study (Table 4), under absorption, the result shows that some compounds exhibited excellent intestinal absorption which confer on them the desired drug‐likeness properties of a compound for oral administration. Hypericin, ursolic acid, and venetoclax (standard anticancer drug) all had excellent intestinal absorption potentials. However, some compounds were found to exhibit poor intestinal absorption. For example, glycyrrhizic acid, crocin, and tubeimoside‐1 all had 0% intestinal absorption potentials. This is a negative attribute if they are to be considered for oral administration. Under distribution, only β‐sitosterol will be able to cross the blood–brain barrier (BBB) (Table 4). This is because only compounds with logBB values greater than 0.3 will be able to cross the BBB. For the ability of compounds to cross the CNS, only compounds with logPS (permeability surface) greater than −2 will be able to do so (Pires et al., 2015). From the result, only β‐sitosterol (−1.705), glabridin (−1.8), and ursolic acid (−1.187) had logPS greater than −2. Under metabolism, it was found that none of the compounds will be a metabolite of CYP2D6 while many are metabolizable by CYP3A4. Under toxicity, none of the compounds would be mutagenic. Finally, few compounds (solanine, tomatidine, and ursolic acid) were found to be hepatotoxic. The two standard Wnt/β‐catenin pathway inhibitors IWP4 and cardionogen 1 are predicted to be hepatotoxic (Table 4).

**TABLE 4:** ADMET properties of front‐runners and standard drugs by pkCMS.

S/N	Selected front‐runners	Absorption			Distribution		Metabolism		Excretion	Toxicity			
		Caco‐2	Int. abs	P‐gp	BBB	CNS	CYP2D6	CYP3A4	TC	Ames	MTD	LD_50_	Htox
1.	Glycyrrhizic acid	−0.812	0	–	−1.494	−4.206	No	Yes	−0.304	No	0.389	2.48	No
2.	Solanine	−0.623	16.89	–	−1.719	−4.745	No	Yes	−0.373	No	−2.677	3.08	Yes
3.	Polyphyllin I	−0.779	51.194	I	−1.857	−4.516	No	Yes	0.365	No	−3.053	3.109	No
4.	Crocin	−1.136	0	I	−2.576	−6.49	No	No	1.701	No	−0.068	2.631	No
5.	Hypericin	−2.735	100	I & II	−1.561	−3.443	No	Yes	0.004	No	0.438	2.482	No
6.	Tubeimoside‐1	−1.204	0	I	−2.667	−5.446	No	No	−0.687	No	0.197	2.486	No
7.	Diosmin	0.305	29.319	–	−1.795	−4.836	No	No	−0.113	No	0.565	2.512	No
8.	Rutin	−0.949	23.446	–	−1.899	−5.178	No	No	−0.369	No	0.452	2.491	No
9.	Baicalin	−0.67	26.224	–	−1.331	−3.811	No	No	0.04	No	0.652	2.634	No
10.	Tomatidine	1.3	91.614	I & II	0.008	−2.738	No	Yes	0.039	No	−0.256	2.491	Yes
11.	Ginkgolide‐C	1.074	39.36	I	−0.629	−3.057	No	No	0.342	No	−0.869	3.082	No
12.	Myricetin	0.095	65.93	–	−1.493	−3.709	No	No	0.422	No	0.51	2.497	No
13.	β‐Sitosterol	1.201	94.464	I & II	0.781	−1.705	No	Yes	0.628	No	−0.621	2.552	No
14.	Glabridin	1.284	94.16	I	0.087	−1.8	No	Yes	0.121	No	−0.395	2.523	No
15.	Ursolic acid	1.171	100	–	−0.141	−1.187	No	Yes	0.083	No	0.199	2.054	Yes
16.	Withanolide	0.831	99.2	I & II	−0.315	−2.696	No	Yes	0.347	No	−0.867	2.831	No
17.	Quercetin	−0.229	77.207	–	−1.098	−3.065	No	No	0.407	No	0.499	2.471	No
Standard drugs													
IWP4		1.334	93.41	I & II	−1.231	−2.203	No	Yes	0.408	No	0.154	2.408	Yes
Cardionogen 1		1.502	96.178	–	0.499	−2.926	No	Yes	0.212	No	−0.045	2.778	Yes
Other anticancer drugs													
Venetoclax		0.847	100	I & II	−1.747	−3.119	No	Yes	−0.096	No	0.278	2.604	Yes
Etoposide		0.403	75.614	I & II	−1.567	−4.115	No	Yes	−0.068	No	0.171	3.25	No
Teniposide		0.312	84.449	I & II	−1.756	−3.9	No	Yes	0.428	No	−0.101	2.624	No
(−)‐Rapamycin		0.567	62.002	I & II	−1.674	−2.941	No	Yes	0.558	No	−0.577	3.285	Yes

*Note*: Caco‐2 permeability (log Papp in 10–6 cm/s).

Abbreviations: BBB permeability, blood–brain barrier permeability (log BB); CNS permeability, Central nervous system permeability (log PS); Int. abs, Intestinal absorption (% absorbed); LD_50_, Lethal dose at 50% inhibition (oral rat acute toxicity (mol/kg)); MRTD, Maximum tolerated dose (human) (log mg/kg/day); P‐gp, P‐glycoprotein inhibitors I or II; TC, Total clearance (log mL/min/kg).

## 4 CONCLUSION

Wnt/β‐catenin signaling pathway is a collection of signal transduction pathways that play a role in development, organogenesis, and embryogenesis as it concerns morphogenesis and cellular movements. The abnormal activation of Wnt/β‐catenin signaling pathway promotes cancer stem cell renewal, proliferation, and differentiation, playing important roles in carcinogenesis and therapeutic response. This study has successfully identified promising bioactive compounds with potentials to inhibit β‐catenin or Wnt/β‐catenin signaling through molecular docking simulation. Among 313 compounds docked, the following compounds glycyrrhizic acid, solanine, polyphyllin I, crocin, hypericin, tubeimoside‐1, diosmin, and rutin came top with better binding energies compared to standard anticancer drugs which make them potential drug candidates. However, from the ADMET study, a few of them were found to be very toxic which calls for caution when considering them as oral drug candidates.

## AUTHOR CONTRIBUTIONS

**Chukwuebuka Egbuna:** Conceptualization (equal); data curation (equal); formal analysis (equal); investigation (equal); methodology (equal); project administration (equal); software (equal); supervision (equal); validation (equal); writing – original draft (equal); writing – review and editing (equal). **Kingsley C. Patrick‐Iwuanyanwu:** Data curation (equal); formal analysis (equal); project administration (equal); validation (equal); writing – original draft (equal); writing – review and editing (equal). **Eugene N. Onyeike:** Conceptualization (equal); data curation (equal); formal analysis (equal); investigation (equal); validation (equal); writing – original draft (equal). **Chukwuemelie Zedech Uche:** Data curation (equal); investigation (equal); resources (equal); software (equal); validation (equal); visualization (equal); writing – original draft (equal); writing – review and editing (equal). **Uchenna Petronilla Ogoke:** Investigation (equal); methodology (equal); project administration (equal); software (equal); visualization (equal); writing – original draft (equal). **Muhammad Riaz:** Data curation (equal); investigation (equal); methodology (equal); validation (equal); writing – original draft (equal). **Ebube Nnamdi Ibezim:** Data curation (equal); formal analysis (equal); methodology (equal); project administration (equal); software (equal); validation (equal); visualization (equal). **Johra Khan:** Data curation (equal); investigation (equal); methodology (equal); project administration (equal); software (equal); validation (equal); writing – original draft (equal); writing – review and editing (equal). **Kamoru A. Adedokun:** Formal analysis (equal); investigation (equal); software (equal); validation (equal). **Sikiru O. Imodoye:** Data curation (equal); methodology (equal); resources (equal); software (equal). **Ibrahim O. Bello:** Formal analysis (equal); project administration (equal); software (equal); visualization (equal). **Chinaza Godswill Awuchi:** Data curation (equal); formal analysis (equal); methodology (equal); project administration (equal); software (equal); supervision (equal); validation (equal); visualization (equal); writing – review and editing (equal).

## CONFLICT OF INTEREST STATEMENT

The authors declare no conflict of interest whatsoever.

## ETHICS STATEMENT

The study does not involve humans or animals.

## CONSENT FOR PUBLICATION

All the authors consent to the publication.

## Data Availability

Additional data will be made available on request.
